# COVID-19 severity: Studying the clinical and demographic risk factors for adverse outcomes

**DOI:** 10.1371/journal.pone.0255999

**Published:** 2021-08-11

**Authors:** Naila Shoaib, Naila Noureen, Rimsha Munir, Farhad Ali Shah, Noshaba Ishtiaq, Nazia Jamil, Rida Batool, Mohammad Khalid, Ihsan Khan, Naser Iqbal, Nousheen Zaidi

**Affiliations:** 1 Cancer Biology Lab, Institute of Microbiology and Molecular Genetics (MMG), University of the Punjab, Lahore, Pakistan; 2 Cancer Research Centre (CRC), University of the Punjab, Lahore, Pakistan; 3 Test Zone Diagnostic Centre, Lahore, Pakistan; 4 Hormone Lab, Lahore, Pakistan; 5 Institute of Microbiology and Molecular Genetics (MMG), University of the Punjab, Lahore, Pakistan; Unaizah College of Pharmacy, Qassim University, SAUDI ARABIA

## Abstract

**Background:**

The primary goal of the presented cross-sectional observational study was to determine the clinical and demographic risk factors for adverse coronavirus disease 2019 (COVID-19) outcomes in the Pakistani population.

**Methods:**

We examined the individuals (n = 6331) that consulted two private diagnostic centers in Lahore, Pakistan, for COVID-19 testing between May 1, 2020, and November 30, 2020. The attending nurse collected clinical and demographic information. A confirmed case of COVID-19 was defined as having a positive result through real-time reverse transcriptase polymerase chain reaction (RT-PCR) assay of nasopharyngeal swab specimens.

**Results:**

RT-PCR testing was positive in 1094 cases. Out of which, 5.2% had severe, and 20.8% had mild symptoms. We observed a strong association of COVID-19 severity with the number and type of comorbidities. The severity of the disease intensified as the number of comorbidities increased. The most vulnerable groups for the poor outcome are patients with diabetes and hypertension. Increasing age was also associated with PCR positivity and the severity of the disease.

**Conclusions:**

Most cases of COVID-19 included in this study developed mild symptoms or were asymptomatic. Risk factors for adverse outcomes included older age and the simultaneous presence of comorbidities.

## 1. Introduction

The coronavirus disease 2019 (COVID-19) pandemic caused by the severe acute respiratory syndrome coronavirus 2 (SARS-CoV-2) has spread globally. As of May 24, 2021, there were **166,346,635** confirmed cases and **3,449,117** deaths, with the numbers still increasing worldwide [1]. In Pakistan, the first case of COVID-19 was reported on February 26, 2020. Since then, the country has seen several changes in the course of the pandemic. Until May 24, 2021, **903,599** confirmed COVID-19 cases, and more than **20 thousand** related deaths have been recorded in Pakistan [2]. The country was initially seen as a very high-risk region for the COVID-19 pandemic because of the less efficient healthcare system and infrastructure. Moreover, implementing nonpharmaceutical interventions such as social-distancing and universal mask-wearing was challenging, if not virtually impossible, in large overcrowded urban areas. Surprisingly, however, Pakistan has done well to contain the pandemic. Nonetheless, the actual reason for the low infection and mortality rate has been a conundrum for the scientific community.

Pakistan, together with its immediate neighbors, India and Bangladesh, constitutes the Indian subcontinent. These three countries are considered similar in terms of genetic makeup, socioeconomic conditions, and culture. However, several COVID-19-related metrics display significant differences when compared among these countries **(S1 Table**). **S1A–S1C Fig** shows the pandemic trend-analysis of new cases, deaths, and test positivity percentage for the three countries.

COVID-19 presents varied clinical features. A recent review estimated that at least one-third of the SARS-CoV-2 infections are asymptomatic [3]. The spectrum of the symptomatic disease also ranges from mild to severe [4–9]. Several studies have focused on understanding the risk factors associated with COVID-19 severity [10]. Age and male gender are the well-established risk factors for severe COVID-19 outcomes [11–22]. Several studies reported that the median age of the hospitalized patients ranged from 49 to 56 years [6, 8, 23]. Moreover, multiple studies show that a disproportionately high number of patients displaying severe symptoms were males [24–26]. Several comorbidities–including diabetes, hypertension, and cardiovascular diseases–have also been associated with severe illness and mortality [11, 13, 15, 18, 19, 21, 27, 28].

There is a paucity of studies reporting patients’ clinical characteristics and outcomes in Pakistan. Most of the available evidence on COVID-19 risk factors and clinical features is based on studies of hospitalized patients. After the recent surge in cases in our neighboring countries–particulary India and Nepal–there is unprecedented urgency to understand who is most at risk of severe outcomes. Understanding the features associated with COVID-19 susceptibility and severity is critical to guide the local health authorities in allocating the available resources more efficiently. This information also helps in avoiding the over-stressing of the already constrained healthcare system. This work’s primary goal was to determine the risk factors for adverse COVID-19 outcomes in the Pakistani population. To the best of our knowledge, this is the most extensive study from Pakistan on this subject.

## 2. Material and methods

### 2.1. Study-population, ethics, and sample collection

This study includes the individuals (n = 6331) that consulted various collection centers of the two private diagnostic centers (*Hormone Lab*, Lahore, and *TestZone Diagnostic Centre*, Lahore) in Lahore, Pakistan, between May 1, 2020, and November 30, 2020, for COVID-19 testing. Lahore is the 2^nd^ largest (1,772 km^2^ [29]) and second most populated (Population: 11,126,285 [29]) city in the largest province of Pakistan i.e. Punjab. The diagnostic centers also provided a home sampling facility to suspected SARS-CoV-2 positive patients. The institutional biosafety committee-Hormone lab approved the study protocol for human subjects. Informed consent was obtained from each study subject before sample collection. The attending nurse also collected the information on symptoms, clinical history, and demographics of each participant at the time of the sample collection. A confirmed case of COVID-19 was defined as having a positive result through real-time reverse-transcriptase–polymerase-chain-reaction (RT-PCR) assay of nasopharyngeal swab specimens. Only laboratory-confirmed cases were included in the analysis. The patients were classified as asymptomatic, severe, or mild cases based on the National Institute of Health [30]. Asymptomatic cases were defined as the individuals that tested positive for SARS-CoV-2 but had no symptoms that are consistent with COVID-19. The mild-symptom cases were classified as the patients who had any of the various signs and symptoms of COVID-19 (e.g., fever, cough, sore throat, malaise, headache, muscle pain, nausea, vomiting, diarrhea, loss of taste and smell) but who do not have shortness of breath, dyspnea, or abnormal chest imaging. Severe cases were defined as adults with clinical signs of pneumonia (fever, dyspnea, cough, and fast breathing) or abnormal chest imaging.

### 2.2. RNA extraction and RT-PCR

Deep nasal cavity swab samples were collected from patients, and viral RNA was extracted by using FavorPrep™ Viral Nucleic Acid Extraction Kit according to the manufacturer’s instructions. SARS‐CoV‐2 was detected by RT‐PCR assay using a COVID‐19 Nucleic Acid Detection Kit according to the manufacturer’s protocol (Sansure Biotech Inc or GeneProof Kit).

### 2.3. Statistical analysis

Data were analyzed *vi*a GraphPad Prism Software, Version VII or MS-Excel using descriptive statistics such as percentages and frequencies. Monthly positive test percentages were calculated using the following equations:

% Positive Tests = (total number of positive cases per month/ total number of tests done per month) x 100

## 3. Results & discussion

Previous studies provide limited evidence on the clinical characteristics of SARS-CoV-2 infection in Pakistan [31–43]. These studies had several limitations, including a small sample size or data collection at a single diagnostic center. For the presented analysis, the data were collected at two major diagnostic facilities with an extensive network of collection centers spread across Punjab–the most populated province in Pakistan (Area: 205,344 km^2^, Population: 109,989,655 [44]). We collected the information on the patient’s age, sex, SARS-CoV-2 PCR status, COVID-19 severity, comorbidities, and the reason for getting tested.

### 3.1. Description of the cohort

**Fig 1:**
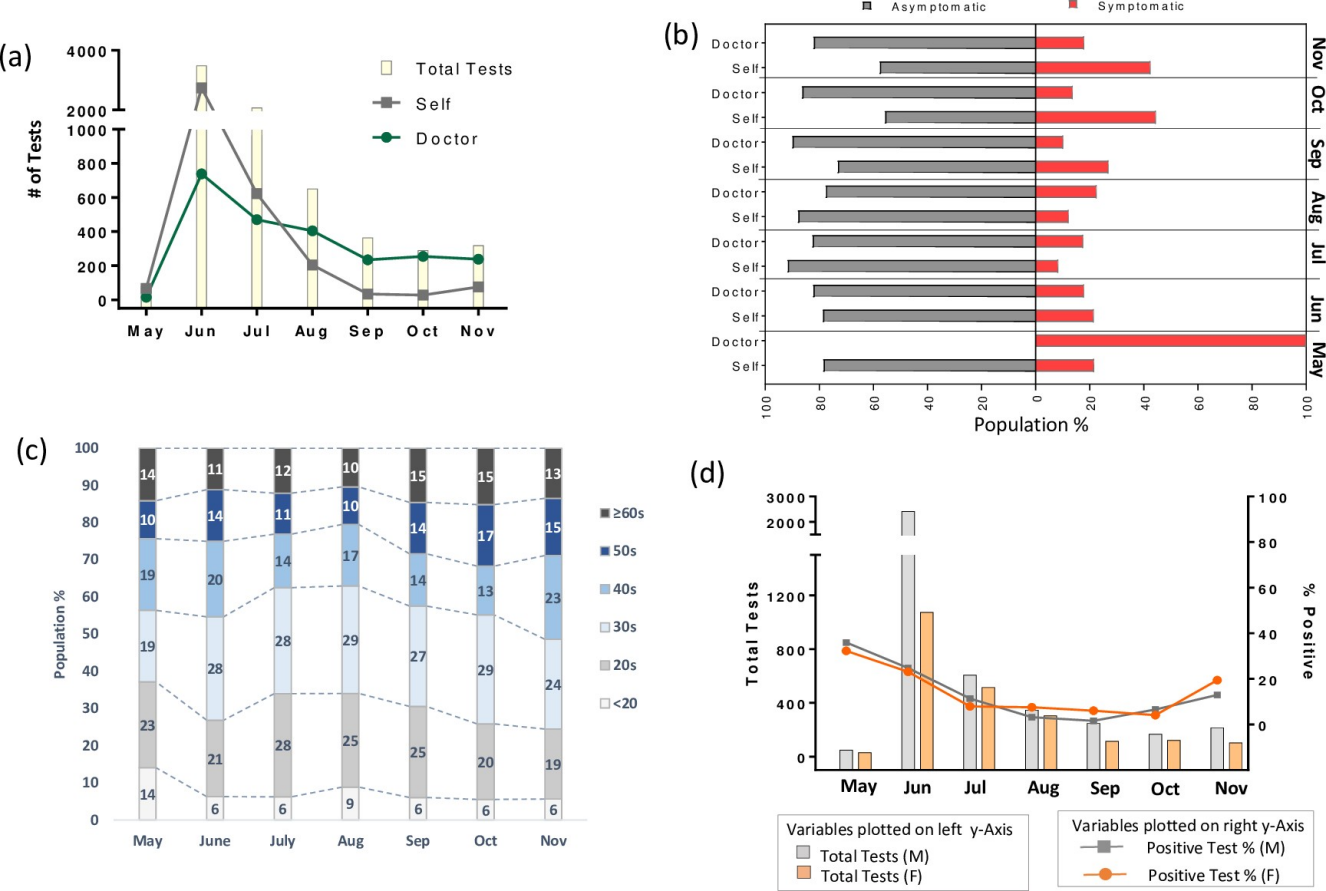
Description of the cohort. **(a)** The total number of tests performed for the presented work upon doctor’s recommendation or self-motivation. **(b)** Percentage of symptomatic and asymptomatic participants reaching out for COVID-19 testing upon doctor’s advice or self-motivation. **(c)** Age-distribution of the cohort **(d)** COVID-19 new tests per thousand people (males or females) (*left y-axis*, vertical lines), and positive test percentage for males and females (*right y-axis*, stacked line) between May 1, 2020, and November 30, 2020.

For the present study, we examined the individuals (n = 6331) that consulted two private diagnostic centers in Lahore for COVID-19 testing between May 1, 2020, and November 30, 2020. The highest number of samples were received in June (n = 3491) (**Fig 1A**). This number gradually decreased for the later months. However, this data does not correspond to the total number of tests that were conducted in Pakistan for the given months (**S2 Fig**). For May, June, and July, the number of individuals coming for COVID-19 tests on doctor’s recommendation was higher than those without doctor’s advice (Self) (**Fig 1A**). From Aug till Nov, this trend was reversed, and there was a significant decline in the number of doctor-recommended tests.

Next, we sought to determine whether there was a difference in people reaching out for the COVID-19 test in terms of symptoms. In general, the number of asymptomatic participants remained higher for each category for the entire study period, except in May, where all the doctor-recommended tests were for symptomatic patients **(Fig 1B)**. From Sep onwards, the proportion of symptomatic self-motivated tests was higher than symptomatic doctor-recommended tests **(Fig 1B)**. Before that, the proportion of symptomatic patients was higher in doctor-recommended tests than self-motivated tests, except in June. The study population’s age profile remained more or less consistent, and the highest percentage of participants were in their 20s or 30s **(Fig 1C).**

For the entire duration of the study, the number of total tests for male participants remained higher than for female participants (**Fig 1D**). The highest number of samples were collected in June. The positive test percentages for both sexes showed a similar trend, i.e., decrease from May till Aug (**Fig 1D**). From Sep onwards, there was another surge in positive test percentages.

Next, the cohort was stratified by PCR positivity status. As shown in **S2 Table,** several differences in demographic and clinical characteristics were noted between the PCR positive and negative groups.

### 3.2. Effects of demographic factors on COVID-19 severity

**Fig 2:**
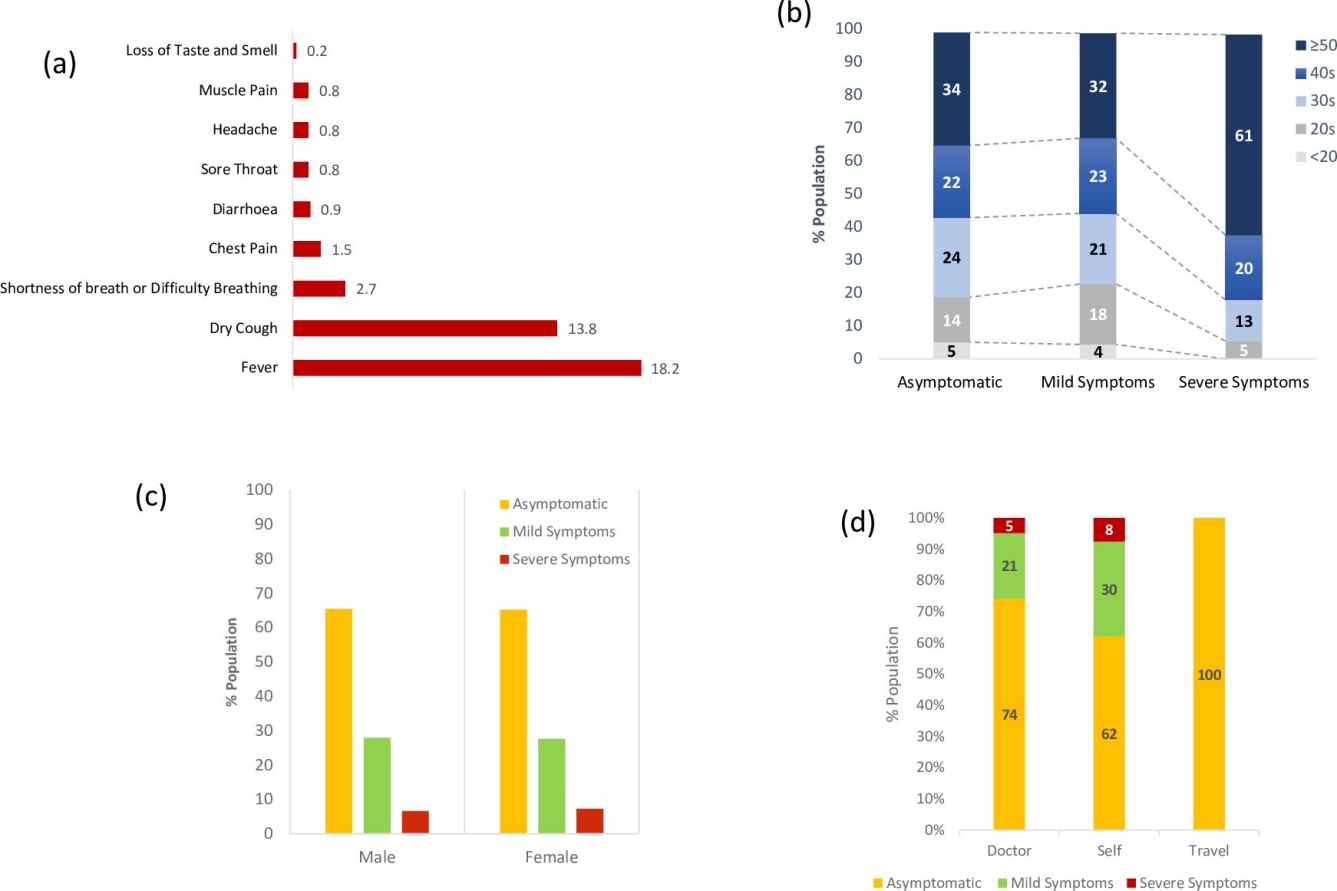
Effects of demographic factors on COVID-19 severity. **(a)** The frequency of various reported symptoms in patients positive for SARS-CoV-2. **(b)** Age-distribution of SARS-CoV-2 positive patients categorized according to their symptoms, i.e., Asymptomatic, with mild or severe symptoms. **(c)** Symptomology in male and female patients **(d)** Symptomology in participants reaching out for COVID-19 test upon doctor’s advice, self-motivation, or as a traveling prerequisite.

**Fig 2A** shows the frequency of reported symptoms in patients positive for SARS-CoV-2. Fever was the most frequent symptom (18.2%), followed by dry cough (13.8%) and shortness of breath (2.7%). The PCR positive group was stratified into three categories, i.e., asymptomatic, mild symptoms, and severe symptoms. We assessed the impact of basic demographic factors, including age and sex, on COVID-19 symptom severity. **S3A Fig** shows the age distribution of the PCR-positive patients. The highest number of individuals were in their 30s and 40s. There was a clear association of symptom severity with age (**Fig 2B**). Our analysis revealed that the highest proportion (61%) of individuals developing severe symptoms were aged 50 or above. Within the severe symptoms group, only 5% of the individuals were in their 20s. The participants below the age of 20 were either asymptomatic or developed only mild symptoms. This group constituted only a small proportion of the SARS-CoV-2 positive group (**S2 Table**). Epidemic data from around the world, including the US [15, 17], China [12, 13], and Italy [16], indicates that higher age is one of the major risk factors for a severe course of COVID-19. A few studies specified the age for increased risk as >64 [17] or >65 [18] years. As mentioned above, for our population, the age of ≥50 was associated with increased risk.

As discussed above, most of the participants opting for COVID-19 testing were males. The SARS-CoV-2 positive group also had a significantly higher number of males (**S2 Table**). When separately assessed, each symptom category comprises more males than females (**S3B Fig)**. However, when male and female populations were separately assessed, the proportion of asymptomatic, mild symptoms, and severe symptoms cases were strikingly similar within each sex category (**Fig 2C**)–with most patients remaining asymptomatic or developing mild symptoms. This contrasts with most other studies showing a strong association between male sex and disease severity [13, 14, 22]. A meta-analysis of 3,111,714 reported global cases has demonstrated that the male patients have almost three times the odds of requiring intensive treatment unit admission and higher odds of death than females [45]. Studies with a large sample size are required to explore this association in the Pakistani population further.

We also recorded why people opted for the COVID-19 tests at the selected diagnostic facilities. The highest proportion (73.4%) of the SARS-CoV-2 positive group opted for the test on the doctor’s recommendation (**S2 Table**). Among the doctor-recommended tests, 74% of the SARS-CoV-2 positive patients were asymptomatic (**Fig 2D**). In contrast, among the self-motivated tests, this number was 61%. A minority of cases (1.5%) opted for the test because it was a prerequisite for traveling abroad (**S2 Table**). All the people opting for the COVID-19 PCR test for traveling purposes claimed to be asymptomatic (**Fig 2D**).

### 3.3. Effects of Comorbidities on COVID-19 severity

**Fig 3:**
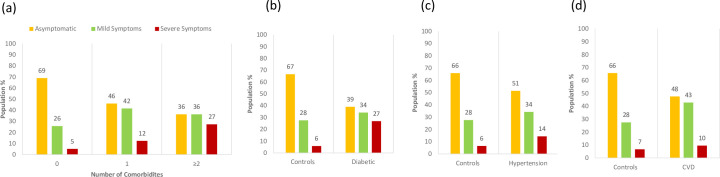
Effects of comorbidities on COVID-19 severity. **(a)** Symptomology in COVID-19 patients classified based on the number of comorbidities. **(b)** Symptomology in COVID-19 patients with diabetes versus the control group. **(c)** Symptomology in COVID-19 patients with hypertension versus the control group. **(d)** Symptomology in COVID-19 patients with cardiovascular diseases versus the control group.

Next, we assessed the impact of comorbidities on the severity of COVID-19. The percentage of individuals with severe symptoms increases with the increasing number of comorbidities **(Fig 3A).** A significantly higher number of diabetic SARS-CoV-2 positive patients developed mild (34%) or severe symptoms (27%) than the control group–non-diabetic SARS-CoV-2 positive patients. For which, these values were 28% and 6%, respectively **(Fig 3B).**

The SARS-CoV-2 positive patients with hypertension also displayed a significantly higher proportion of severe symptom cases **(Fig 3C)**. In addition, the CVD patients showed an increase in the proportion of individuals that developed severe symptoms; however, this trend was comparatively less pronounced **(Fig 3D)**. These data are in line with several previous studies from around the globe, according to which the most vulnerable groups for the poor outcome from COVID-19 are patients with hypertension [11, 13, 15, 19, 21, 27] and diabetes [11, 18, 19, 21, 27, 28], followed by the cardiovascular disease [11, 13, 19]. Previous studies [26, 46, 47] have also shown that diabetes and hypertension are more common among those infected with SARS-CoV-2 than the control groups. Here, we did not observe this phenomenon (**S2 Table**).

The presented work comprehensively examined the effects of several demographic and clinical risk factors associated with COVID-19 severity in Pakistani patients. Our data shows that most cases of COVID-19 developed mild symptoms or were asymptomatic. Risk factors for adverse outcomes included older age and the simultaneous presence of comorbidities. Understanding these risk factors may help health care professionals develop improved clinical management plans based on risk stratification. It may also help in guiding the local health authorities in allocating the available resources more efficiently.

## Supporting information

S1 Table Comparison of population demographics and COVID-19 metrics between India, Pakistan, and Bangladesh. (DOCX)

S2 Table Demographic characteristics of the cohort classified based on the appearance of symptoms. (DOCX)

S1 Fig Trend anlzysis for (a) daily new confirmed COVID-19 cases (per 1M) (b) daily new confirmed COVID-19 deaths (per 1M) & (c) daily percentage of positive COVID-19 tests in Pakistan, India and Bangladesh. Data Source: COVID-19 Data Explorer https://ourworldindata.org.
(PPTX)

S2 Fig The total number of tests performed in Pakistan during the given months. Data Source: https://ourworldindata.org/coronavirus-testing.
(PPTX)

S3 Fig (a) The age distribution of the SARS-CoV-2 positive patients analyzed for the presented study. (b) The total number of male and female SARS-CoV-2 positive patients categorized according to their symptoms, i.e., Asymptomatic, with mild or severe symptoms.
(PPTX)
