# Supplementary material for: COVID-19 severity: Studying the clinical and demographic risk factors for adverse outcomes
Source: PLoS One. 2021 Aug 11;16(8):e0255999. doi: 10.1371/journal.pone.0255999 (PMC8357125; doi:10.1371/journal.pone.0255999)
Supplement: S1 Table — (DOCX) [file pone.0255999.s001.docx]

| **S1 Table.** Comparison of population demographics and COVID-19 metrics between India, Pakistan, and Bangladesh. **Data Source:** Our World in Data -COVID-19 Data Explorer [1]. | | | |
| --- | --- | --- | --- |
|  | **India** | **Pakistan** | **Bangladesh** |
| **Population** | 1.38B | 220.89M | 164.69M |
| **Population density (People per km^2^)** | 450.42 | 255.57 | 1265.04 |
| **Median Age** | 28.2 | 23.5 | 27.5 |
| **Share aged 65+** | 5.99% | 4.5% | 5.1% |
| **Share aged 70+** | 3.41% | 2.78% | 3.26% |
| **GDP per Capita (int.-$)** | 6,427$ | 5,035$ | 3,524$ |
| **Population in Extreme Poverty** | 21.2% | 4% | 14.8% |
| **Human Development Index** | 0.65 | 0.56 | 0.63 |
| **Hospital beds (per 1000)** | 0.53 | 0.6 | 0.8 |
| **Stringency Index** | 81.94 | 64.34 | 83.33 |
| **Life Expectancy** | 69.66 | 67.27 | 72.59 |
| **Total Vaccinations** | 186.41M | 3.84M | 9.64M |
| **Total Vaccinations (per 100)** | 13.51 | 1.74 | 5.85 |
| **New Vaccinations** | 1.48M | 73,712 | 32,265 |
| **New Vaccinations (per 100)** | 0.11 | 0.03 | 0.02 |
| **Total Deaths** | 291,331 | 20,089 | 12,284 |
| **Total Deaths (per 1M)** | 211.11 | 90.94 | 74.59 |
| **New Deaths** | 4,144.86 | 100.71 | 29.71 |
| **New Deaths (per 1M)** | 3 | 0.46 | 0.18 |
| **Total Cases** | 26.03M | 893,461 | 785,194 |
| **Total Cases (per 1M)** | 18,863.7 | 4,044.78 | 4,767.73 |
| **Daily New confirmed COVID-19 Cases** | 283,597.43 | 2,891.57 | 929.57 |
| **Daily New confirmed COVID-19 Cases (per 1M)** | 205.5 | 13.09 | 5.64 |
| **Total Tests** | 313.02M | 12.48M | 5.7M |
| **Total Tests (Per 1K)** | 226.82 | 56.5 | 34.59 |
| **Daily new COVID-19 tests** | 1.8M | 36,049 | 10,975 |
| **Daily new COVID-19 tests per 10,00 people** | 1.3 | 0.16 | 0.07 |
| **Case Fatality rate** | 1.12% | 2.25% | 1.56% |

1. COVID-19 Data Explorer-Our World in Data. <https://ourworldindata.org/explorers/coronavirus-data-explorer?zoomToSelection=true&time=2020-03-01..latest&pickerSort=asc&pickerMetric=population&Metric=Confirmed+cases&Interval=7-day+rolling+average&Relative+to+Population=true&Align+outbreaks=false&country=PAK~BGD~IND>
