# Supplementary material for: COVID-19 severity: Studying the clinical and demographic risk factors for adverse outcomes
Source: PLoS One. 2021 Aug 11;16(8):e0255999. doi: 10.1371/journal.pone.0255999 (PMC8357125; doi:10.1371/journal.pone.0255999)
Supplement: S2 Table — (DOCX) [file pone.0255999.s002.docx]

| **S2 Table.** Demographic characteristics of the cohort classified based on the appearance of symptoms. | | | |
| --- | --- | --- | --- |
| **COVID-19 positive patients Variable** | **Asymptomatic (n=535)** | **Mild Symptoms (n=228)** | **Severe Symptoms (n=57)** |
| **Age (years), median (IQR)** | 42 (30-55) | 40 (28-55) | 53 (45-60) |
| **Gender n (%)** | | | |
| Men | 365 (68.2%) | 156 (68.4%) | 38 (66.7%) |
| Women | 170 (31.8) | 72 (31.6%) | 19 (33.3%) |
| **Reason of test** | | | |
| Doctor Recommended | 138 (25.8%) | 38 (16.7%) | 10 (17.5%) |
| Self | 387 (72.3%) | 190 (83.3%) | 47 (82.5%) |
| Travelling Purpose | 10 (1.9%) | 0 | 0 |
| **Travel History (last 4 wks)** | | | |
| Local | 31 (5.8%) | 20 (8.8%) | 2 (3.5%) |
| Foreign | 5 (0.9%) | 2 (0.9%) | 0 |
| No History | 499 (93.3%) | 206 (90.4%) | 55 (96.5%) |
| **Number of Comorbidities** | | | |
| 0 | 482 (90.1%) | 179 (78.5%) | 37 (64.9%) |
| 1 | 41 (7.7%) | 37 (16.2%) | 11 (19.3%) |
| ≥2 | 12 (2.2%) | 12 (5.3%) | 9 (15.8%) |
| **Type of Comorbidities** | | | |
| Kidney Disease | 5 (0.9%) | 1 (0.4%) | 1 (1.8%) |
| Diabetes | 16 (3.0%) | 14 (6.1%) | 11 (19.3%) |
| BP/Hypertension | 18 (3.4%) | 12 (5.3%) | 5 (8.8%) |
| Cancer | 1 (0.2%) | 0 | 0 |
| Cardiovascular Disease | 10 (1.9%) | 9 (3.9%) | 2 (3.5%) |
| Pulmonary Disease | 2 (0.4%) | 4 (1.8%) | 6 (10.5%) |
| Others | 19 (3.6%) | 24 (10.5%) | 6 (10.5%) |
