# Supplementary material for: COVID-19 severity: Studying the clinical and demographic risk factors for adverse outcomes
Source: PLoS One. 2021 Aug 11;16(8):e0255999. doi: 10.1371/journal.pone.0255999 (PMC8357125; doi:10.1371/journal.pone.0255999)
Supplement: S1 Fig — Trend anlzysis for (a) daily new confirmed COVID-19 cases (per 1M) (b) daily new confirmed COVID-19 deaths (per 1M) & (c) daily percentage of positive COVID-19 tests in Pakistan, India and Bangladesh. Data Source: COVID-19 Data Explorer https://ourworldindata.org. (PPTX) [file pone.0255999.s003.pptx]

## Slide 1
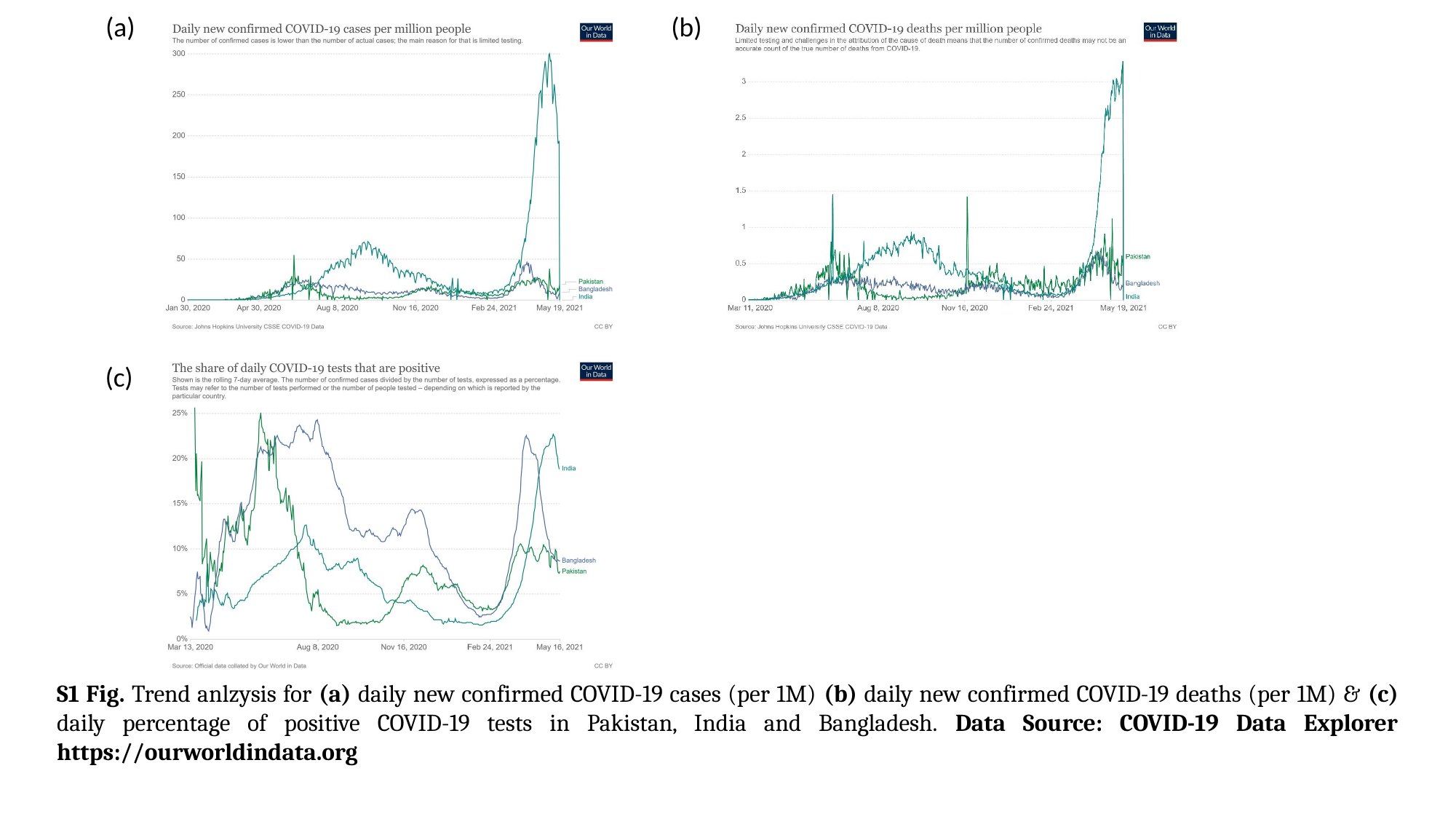

(a)
(b)
(c)
S1 Fig. Trend anlzysis for (a) daily new confirmed COVID-19 cases (per 1M) (b) daily new confirmed COVID-19 deaths (per 1M) & (c) daily percentage of positive COVID-19 tests in Pakistan, India and Bangladesh. Data Source: COVID-19 Data Explorer https://ourworldindata.org
