# Supplementary material for: COVID-19 severity: Studying the clinical and demographic risk factors for adverse outcomes
Source: PLoS One. 2021 Aug 11;16(8):e0255999. doi: 10.1371/journal.pone.0255999 (PMC8357125; doi:10.1371/journal.pone.0255999)
Supplement: S2 Fig — Data Source: https://ourworldindata.org/coronavirus-testing. (PPTX) [file pone.0255999.s004.pptx]

## Slide 1
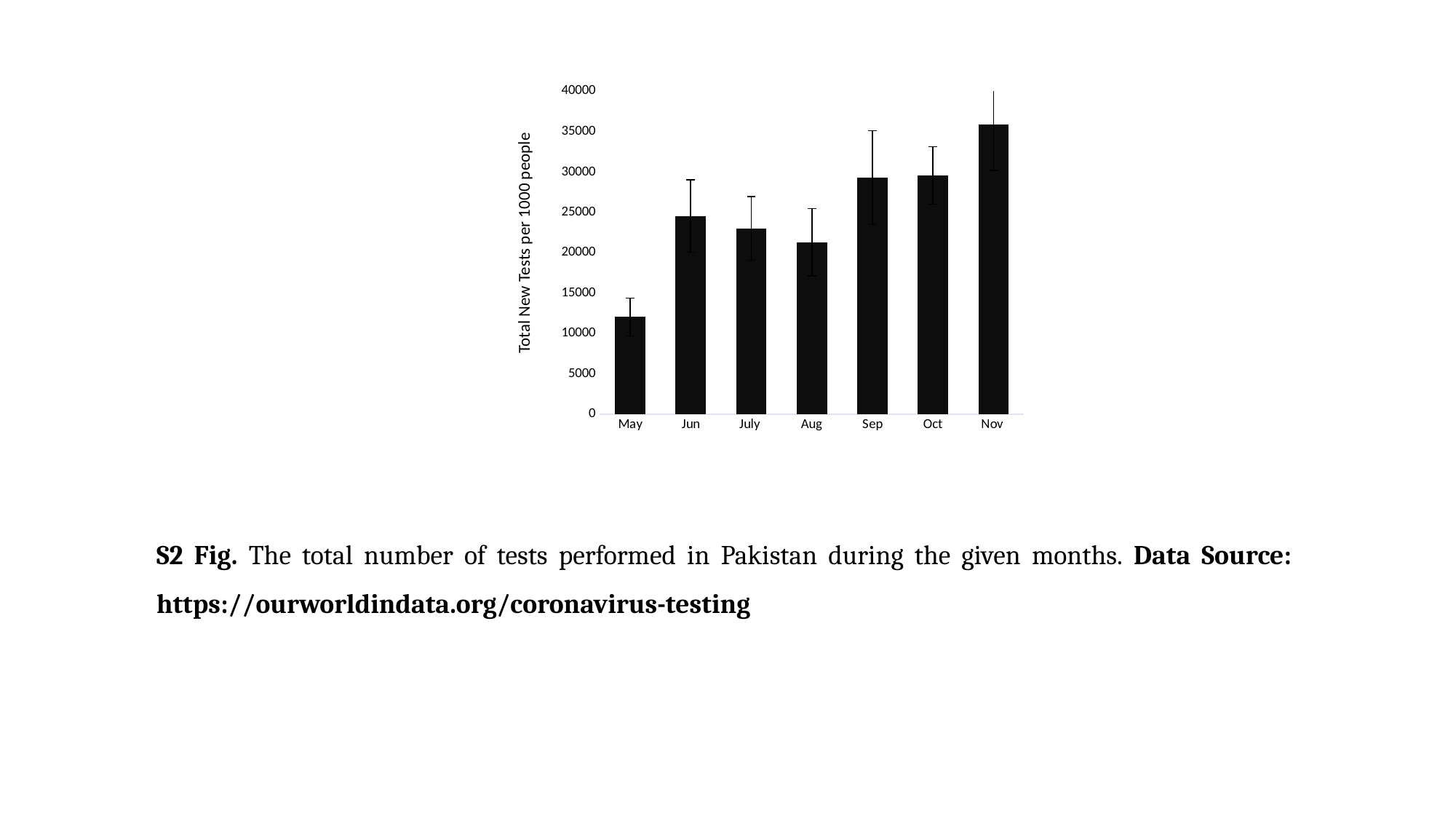

### Chart
| Category | |
|---|---|
| May | 12028.064516129032 |
| Jun | 24535.4 |
| July | 23004.833333333332 |
| Aug | 21284.785714285714 |
| Sep | 29302.566666666666 |
| Oct | 29580.25806451613 |
| Nov | 35919.5 |Total New Tests per 1000 people
S2 Fig. The total number of tests performed in Pakistan during the given months. Data Source: https://ourworldindata.org/coronavirus-testing
