# Supplementary material for: COVID-19 severity: Studying the clinical and demographic risk factors for adverse outcomes
Source: PLoS One. 2021 Aug 11;16(8):e0255999. doi: 10.1371/journal.pone.0255999 (PMC8357125; doi:10.1371/journal.pone.0255999)
Supplement: S3 Fig — (a) The age distribution of the SARS-CoV-2 positive patients analyzed for the presented study. (b) The total number of male and female SARS-CoV-2 positive patients categorized according to their symptoms, i.e., Asymptomatic, with mild or severe symptoms. (PPTX) [file pone.0255999.s005.pptx]

## Slide 1
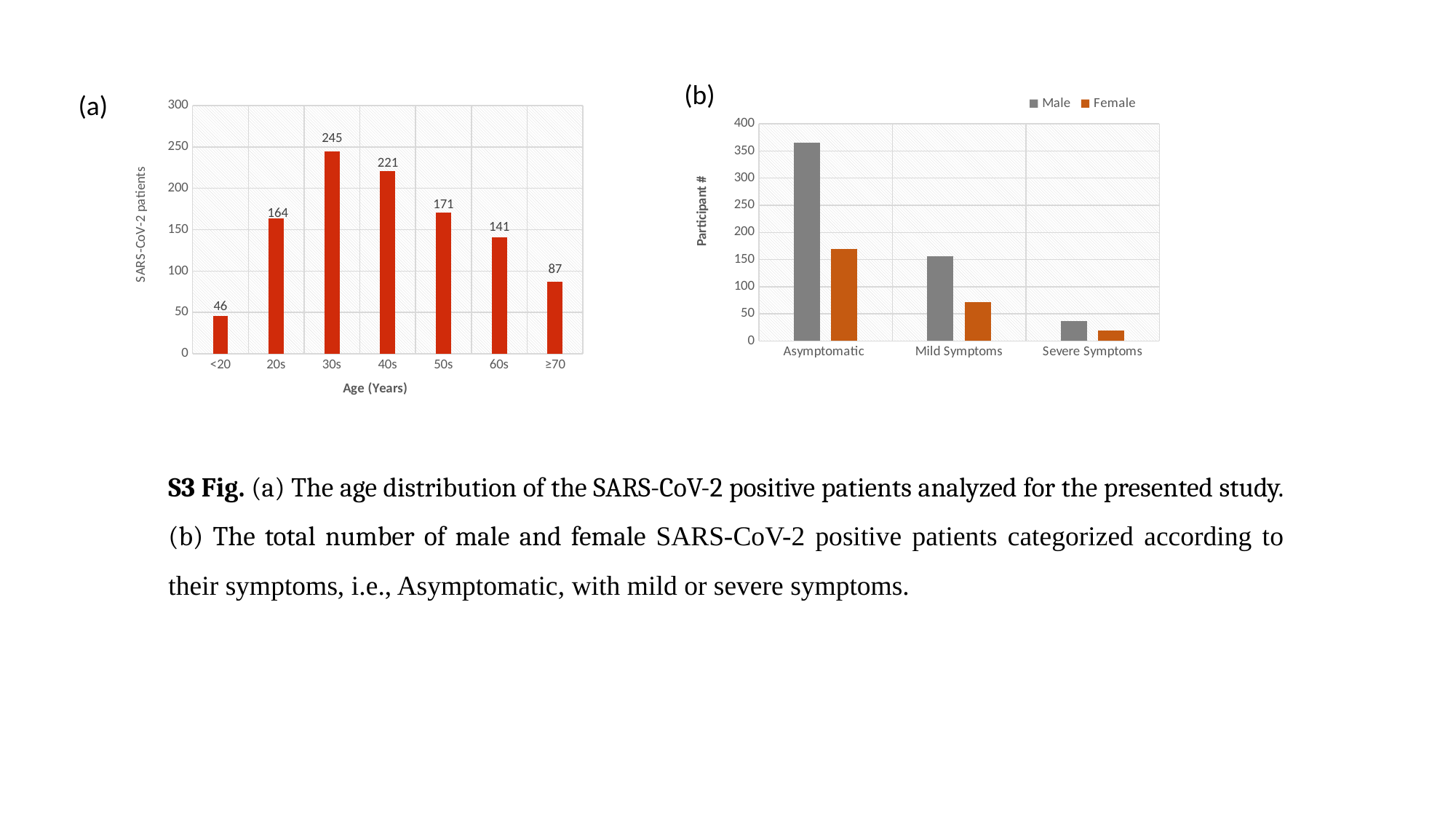

(b)
(a)
### Chart
| Category | Male | Female |
|---|---|---|
| Asymptomatic | 365.0 | 170.0 |
| Mild Symptoms | 156.0 | 72.0 |
| Severe Symptoms | 37.0 | 19.0 |
### Chart
| Category | |
|---|---|
| <20 | 46.0 |
| 20s | 164.0 |
| 30s | 245.0 |
| 40s | 221.0 |
| 50s | 171.0 |
| 60s | 141.0 |
| ≥70 | 87.0 |S3 Fig. (a) The age distribution of the SARS-CoV-2 positive patients analyzed for the presented study. (b) The total number of male and female SARS-CoV-2 positive patients categorized according to their symptoms, i.e., Asymptomatic, with mild or severe symptoms.
